# *Bacillus subtilis* Cell Differentiation, Biofilm Formation and Environmental Prevalence

**DOI:** 10.3390/microorganisms10061108

**Published:** 2022-05-27

**Authors:** Yuxuan Qin, Leticia Lima Angelini, Yunrong Chai

**Affiliations:** 1 Key Laboratory of Biology and Genetic Improvement of Horticultural Crops of the Ministry of Agriculture, Sino-Dutch Joint Laboratory of Horticultural Genomics, Institute of Vegetables and Flowers, Chinese Academy of Agricultural Sciences, Beijing 100081, China; 2 Department of Biology, Northeastern University, Boston, MA 02115, USA; limaangelini.l@northeastern.edu

**Keywords:** *Bacillus subtilis*, biofilm formation, cell differentiation

## Abstract

*Bacillus subtilis* is a soil-dwelling, spore-forming Gram-positive bacterium capable of cell differentiation. For decades, *B. subtilis* has been used as a model organism to study development of specialized cell types. In this minireview, we discuss cell differentiation in *B. subtilis*, covering both past research and recent progresses, and the role of cell differentiation in biofilm formation and prevalence of this bacterium in the environment. We review *B. subtilis* as a classic model for studies of endospore formation, and highlight more recent investigations on cell fate determination and generation of multiple cell types during biofilm formation. We present mechanistic details of how cell fate determination and mutually exclusive cell differentiation are regulated during biofilm formation.

## 1. Introduction

Bacteria are primed to respond and adapt to changes in their living environments in a timely fashion. The adaptation often involves complex processes where cells differentiate into phenotypically distinct cell types to better suit the environments. Cell differentiation has been studied in different model bacteria. For example, the Gram-positive bacterium *Bacillus subtilis* has been used as a model organism for decades to study bacterial spore formation under nutrient starvation [1]. Under similar nutrient-limited conditions, *Myxococcus xanthus* forms fruiting bodies, structured aggregates consisting of millions of differentiated cells including arthrospores, to cope with nutrient stress in the environment [2]. Under nitrogen-limiting conditions, some cyanobacterial species such as *Anabeana* sp. can grow in filaments, and about 5–10% of the cells per filament differentiate into heterocysts that are specialized nitrogen-fixing cells [3]. More recent studies suggest that cell differentiation frequently occurs in a multicellular community, and as a result, multiple distinct cell types coexist in the community. This phenomenon is often termed division-of-labor to highlight the collaborative nature among differentiated cell types in performing multitasks for the community [4]. This also raises new questions about how multiple cell differentiation events occur in parallel and how generations of distinct cell types are regulated during formation of the multicellular communities, or biofilms.

Cell differentiation is perceived as a bacterial strategy for more efficient proliferation or better survival of the population in adaptation to changing environments, benefiting from division-of-labor by individual cells in the community. Coexistence of multiple cell types provide an advantage to the population in the natural environment. When confronting unexpected environmental fluctuations, a highly differentiated heterogeneous population allows expression of a diverse set of genes in different cell types, and thus permits high optimization of resources available to the bacterial community. Maintaining a heterogeneous population also provides evolutionary advantages. Under extreme stresses, all but one or a few specific cell types might survive while others die. Coexistence of distinct cell types is thus considered a bet-hedging strategy [5].

*B. subtilis* is regarded as an excellent model organism to study bacterial cell differentiation and development. Research in the past several decades has explicated cell differentiation programs such as genetic competence and sporulation, and their underlying molecular mechanisms. The knowledge gained has greatly contributed to the understanding of general bacterial genetics, physiology, development, and signaling. However, those studies were typically carried out in test tubes using domesticated laboratory strains. Recent studies have shown that cell differentiation in *B. subtilis* could differ significantly between laboratory settings and natural soil environments, between domesticated strains and wild isolates, and between free-living population and biofilm communities. Therefore, it is worth taking a closer look at these well-studied cell differentiation programs under conditions that better resemble their living conditions in the natural environments. This will allow us to gain comparative information about both shared features and variations of these developmental programs under different conditions. In this review, we discuss classic examples of cell differentiation in *B. subtilis* as well as highlight the biofilm community as a new platform to understand complex and parallel cell differentiation processes intricately linked to biofilm development.

## 2. *B. subtilis* Is a Classic Model to Study Cell Differentiation

Historically, *B. subtilis* served as a model system to study bacterial development, cell signaling, and gene regulation [1,6,7,8]. *B. subtilis* is also well-known for cell differentiation. At the onset of stationary phase, a small subpopulation of cells can differentiate into competent cells that are capable of taking up free DNA from the environment, as a way to increase genetic diversity. In *B. subtilis,* the complex regulatory network controlling competence and differentiation has been elegantly elucidated [6]. *B. subtilis* can also differentiate, through a complex process, into dormant spores that are highly resistant to various environmental stresses [1]. Built upon past several decades of research, a substantial amount of knowledge has been gained on how *B. subtilis* cells differentiate, and the underlying molecular mechanisms of this process.

**Figure 1:**
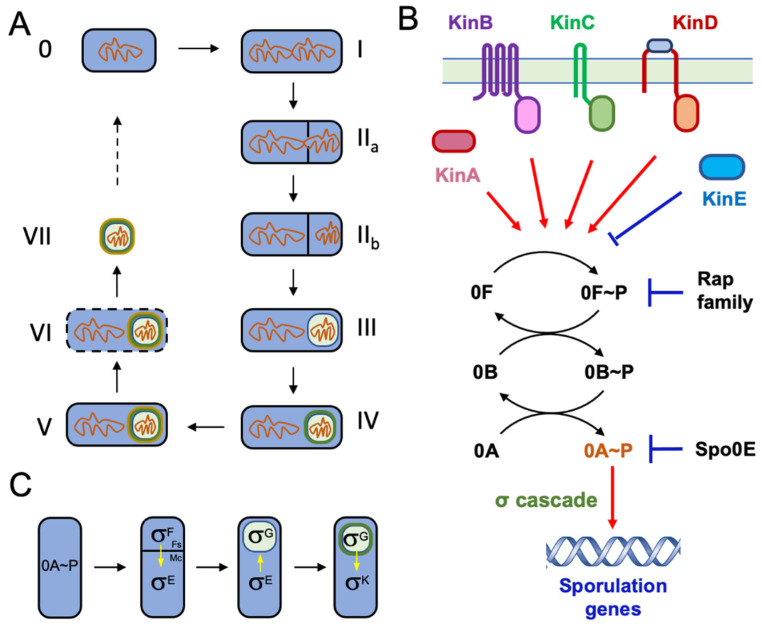
**Diagram of spore formation and gene regulation network for sporulation in *B. subtilis.*** (**A**) Diagram of spore formation. Shown are different stages of sporulation in *B. subtilis* from stage 0 to stage VII. Stage 0: vegetative cell; stage I: genome replication; stage II: asymmetric division (stage II is shown in two different substages, one with formation of asymmetric septum (IIa) and the other with the completion of asymmetric division (IIb)); stage III: forespore engulfment; stage IV: spore cortex formation; stage V: spore coat formation; stage VI: mother cell lysis; stage VII: phase-bright free spore. (**B**) Signal transduction and phosphorelay for activation of sporulation in *B. subtilis*. The three membrane-associated sporulation kinases (KinB, KinC, and KinD) and the cytoplasmic histidine kinase KinA act together in activating sporulation by sensing a diverse set of environmental and cellular signals. KinD has an extracellular CACHE domain involved in direct sensing of plant root-released l-malic acid [16]. KinE is proposed to act as a phosphatase rather than a kinase under normal sporulation conditions [17]. Spo0F (0F), Spo0B (0B), and Spo0A (0A) constitute the phosphorelay. High levels of phosphorylated Spo0A (0A~P) directly and indirectly activate hundreds of genes involved in sporulation, some of which are through mother cell or forespore-specific sigma factors that function in a cascade. The phosphatase Spo0E negatively regulates Spo0A~P and the Rap family phosphatases negatively regulate Spo0F~P. (**C**) Increased Spo0A~P levels lead to activation of the first sporulation sigma factor F (σ^F^) in the forespore (Fs), followed by sequential activation of several other alternative sigma factors (σ^E^, σ^G^, and σ^K^) in either the mother cell (Mc) or the forespore compartment [12].

The discovery that *Bacillus* species are capable of forming endospores can be traced back to almost a century and half ago when it was first documented by Cohn and Koch in 1876 [9,10]. Nowadays, the multiple stages (0 to VII) of endospore development have been described in detail based on morphological distinction and application of cell biology approaches (Figure 1A) [1,11]. Hundreds of genes involved in endospore formation have been characterized and categorized based on their functions in specific sporulation stages [12]. Differentiation into spores is triggered by nutrient starvation. Several sporulation kinases sense various environmental or cellular signals linked to nutrient status, and collectively activate the sporulation master regulator Spo0A through protein phosphorylation (Spo0A~P), either directly or via a phosphorelay (Figure 1B) [13]. A number of phosphatases (Spo0E and the Rap family) are also involved in modulating the phosphorelay and Spo0A phosphorylation levels, providing a way for integration of multiple signals in this decision-making process [13]. Rising Spo0A~P activities lead to activation of the first sporulation sigma factor F (σ^E^) in the forespore, followed by sequential activation of several other alternative sigma factors (σ^E^, σ^G^, and σ^K^) in either the mother cell or the forespore compartment (Figure 1C). Each sigma factor controls a set of genes that function during specific sporulation stages [12]. The genetic pathway governing sporulation in *B. subtilis* can be better described as a temporally and spatially orchestrated expression of a network of genes. Even though studies to characterize genes important for sporulation have been carried out for several decades, new sporulation genes are being identified with novel techniques and sometimes under altered sporulation conditions [14].

The process of endospore formation after initiation is irreversible, energy-consuming, and takes hours to be completed. Interestingly, a recent study showed that not all spores are made the same [15]. During the sporulation life cycle, there seems to be a divided choice favoring either the quantity, or the quality, of spores, or a tradeoff between the quality and quantity of sporulation, depending on different nutrient conditions. In other words, spores could be made at a high yield but have relatively poor quality, and vice versa. The same study further shows that the quality strategy is favored when spore revival (germination) is triggered by poor nutrients. It will be interesting to learn whether this strategy has a significant impact on the fitness of the *B. subtilis* population in the natural environment, and how this well-studied cell differentiation program differs in response to varying environmental conditions.

## 3. Biofilm Is a New Platform to Study *B. subtilis* Cell Differentiation

**Figure 2:**
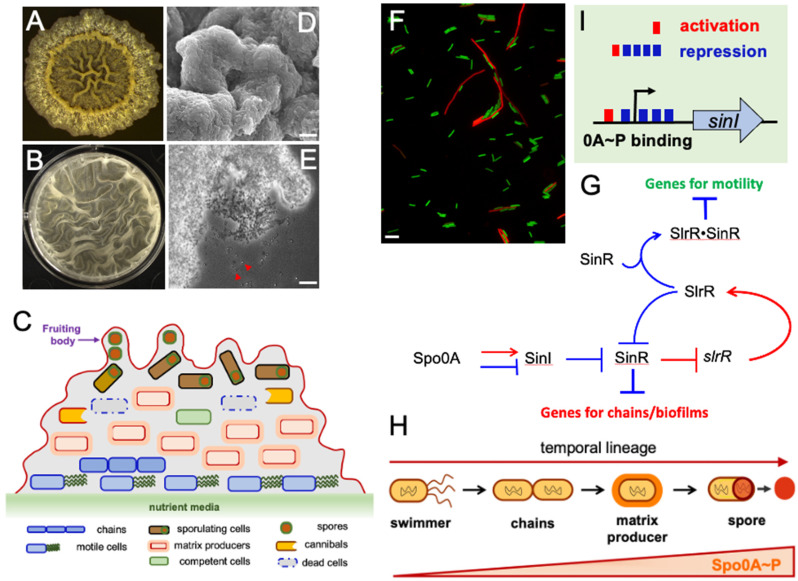
***B. subtilis* cell differentiation and mutually exclusive regulation in a multicellular community.** (**A**,**B**) Colony (**A**) and pellicle (**B**) biofilms formed on a solid agar medium or at the air–liquid interface by *B. subtilis*. (**C**) A cartoon demonstrating the coexistence of multiple differentiated cell types in a *B. subtilis* biofilm. A fruiting body is an aerial projection rising from the surface of the biofilm. Spores are preferentially located at the tip of the fruiting bodies. (**D**) A scanning electron microscopy image of the fruiting body structure. Scale bar, 5 μm. (**E**) A bright-field image of free spores associated with the tip of the fruiting body from a thin-section sample of a biofilm (images are provided by Angelini L). Arrows point to phase-bright spores. Scale bar, 25 μm. (**F**) Differentiation of two mutually exclusive cell types, motile and sessile chained cells, in a population. Motile cells are producing GFP under the control of the promoter for the motility gene *hag* while chained cells are producing mKate2 under the control of the promoter for the biofilm matrix gene *tapA*. Scale bar, 5 μm. (**G**) The epigenetic switch controlling the two mutually exclusive cell types in (**F**) consists of primarily three regulatory proteins, SinR, SinI, and SlrR. SinR is a biofilm repressor, Sin I is an antagonist protein of SinR whose gene is activated by Spo0A~P (0A~P). SlrR is another SinR counteracting protein. SlrR and SinR form a double negative feedback loop in which SinR represses the gene *slrR* while the SlrR protein antagonizes SinR, similar to SinI. A heterocomplex of SinR•SlrR represses motility and cell separation genes [24]. (**H**) A temporal lineage of *B. subtilis* cell development. 0A~P levels are a key determinant of cell differentiation. Intermediate levels of 0A~P drive the transition of motile cells to chains of cells also expressing matrix genes and shut down motility genes indirectly through the SinI-SinR-SlrR epigenetic switch [24]. High levels of 0A~P activate hundreds of genes involved in sporulation, thus leading some matrix producers to become sporulating cells while simultaneously turning off matrix production. (**I**) The regulatory region of *sinI* contains both an activator and multiple operators of 0A~P, whose sequences imperfectly match the consensus 0A~P binding site. This allows both activation of *sinI* by intermediate levels of 0A~P and repression of *sinI* when the 0A~P concentration reaches high levels [35].

Biofilms are structurally complex multicellular communities of microbial cells encased by a self-produced matrix [18]. Undomesticated strains of *B. subtilis* are capable of forming robust biofilms under different laboratory settings (Figure 2A,B). This ability appears to have been lost in domesticated legacy strains of *B. subtilis*, which have been used for genetic studies in the laboratory for many decades [19]. Since the late 1990s, researchers started utilizing *B. subtilis* as a model organism to study biofilms. To date, there is a huge collection of published studies on the biofilms of this bacterial species.

Cell differentiation is a hallmark feature during bacterial biofilm development [20]. These two processes are intricately linked to each other. Thus, biofilms provide an excellent platform to study cell differentiation in a more spatially and temporally interactive context. Further, some of the mutually exclusive regulations governing cell differentiation can only be observed during multicellular development. Cells in the *B. subtilis* biofilms have demonstrated a multitude of distinct types (Figure 2C) [21]. At the genetic level, cell differentiation is primarily a result of heterogeneity in gene regulation in a population of genetically identical cells when they respond to environmental cues. Previous studies have explored molecular mechanisms of gene regulation heterogeneity in controlling cell differentiation in the biofilm or other multicellular structures formed by *B. subtilis* [21,22].

## 4. “Explorers” versus “Settlers”

In both a population of planktonic *B. subtilis* cells and the biofilm, clear distinction of two morphologically different cell types can be observed: those highly motile singlets and doublets vs. those sessile chains of cells (Figure 2F) [23]. The former consists of the majority of a growing population (~90–95%). Studies based on gene-specific fluorescent reporters show that these singlets and doublets are strongly expressing motility and chemotaxis genes: they are “explorers”. The latter subpopulation, often less abundant (~5–10%), is composed of sessile cells, in which genes needed for motility, chemotaxis, and cell separation are shut down, while genes whose functions contribute to a sessile life style are turned on [24,25]. These sessile cells are considered “settlers”. It is plausible to speculate that the coexistence of such two subpopulations is a bet-hedging strategy that allows individual cells in the population to be able to both occupy the current niche and explore new territories. Both subpopulations of cells are also found in a biofilm although with altered ratios and spatial locations overtime during biofilm development [26]. These two cell types are mutually exclusive, which is not only reflected in cell morphology and functionality, but also on gene regulation (Figure 2G) [24,27]. A three-protein regulatory system (SinI-SinR-SlrR) regulates the genes for motility, chemotaxis, and cell separation, and those whose functions are related to biofilm formation inversely [24,25]. The control on cells in the sessile state (chains) is reinforced by an epigenetic switch because of the memory and ultra-sensitivity of SinI-SinR-SlrR-mediated feedback regulations [24,25,28]. Additional players such as the YmdB protein were also shown to be involved in the above regulation [29].

## 5. Matrix Producers versus Spore Formers

The master regulator Spo0A governs cell development and generation of multiple cell types in *B. subtilis.* Spo0A is essential for both sporulation and biofilm formation [30,31]. Upon sensing biofilm-inducing signals in the environment, the same set of histidine kinases initiates a signal transduction cascade that ultimately leads to increased Spo0A~P levels (Figure 1B). This implies that the program for sporulation is embedded in the *B. subtilis* biofilm development, likely for important evolutionary reasons, an idea that we will further discuss below. Although the same triggers and signal transduction pathways are thought to be involved in both biofilm formation and sporulation, contributions by each of the signal-sensing histidine kinases differ noticeably between the two programs [17,32]. For example, KinA was shown to be critical for initiation of sporulation but much less important for induction of biofilm formation. The opposite is true for KinC, another histidine kinase [32,33].

When cells are grown under laboratory settings, Spo0A~P concentrations gradually rise via protein phosphorylation [31]. Spo0A~P levels demonstrate a broad heterogeneity in individual cells in the population [34]. In a subpopulation of cells, Spo0A~P reaches an intermediate level sufficient to activate *sinI*, a key regulatory gene for biofilm induction (Figure 2G) [35]. *sinI* encodes a small protein that counteracts the biofilm repressor SinR [36]. SinI, together with another antagonist, SlrR, acts on SinR to trigger de-repression of SinR-controlled biofilm matrix operons [24]. To this point, this subpopulation of cells differentiates into matrix producers. SlrR also forms a self-reinforcing double negative feedback loop with the repressor SinR. Consequently, matrix-producing and non-producing cells demonstrate a sharp bimodal distribution in the biofilm [24,27]. Interestingly, the secreted matrix is shared by the entire community, another example of division-of-labor [37]. A recent study suggests that even among the matrix-producing cells, cells can be further distinguished in producing the two matrix components (EPS and TasA) differentially; there are cells that only produce EPS and those that produce both EPS and TasA [37]. This study highlights how cell differentiation can occur and cause division-of-labor even in the same functional group of cells.

Some matrix producers in the *B. subtilis* biofilm undergo further development into sporulation [26]. Spores often constitute 15–20% of the total cell population in a mature biofilm [28]. It has been proposed that levels of Spo0A~P are a key determinant of the cell fate. Intermediate levels of Spo0A~P induce cells to produce matrix [35]. When Spo0A~P levels keep rising in those cells and eventually reach a threshold sufficient to activate hundreds of genes involved in sporulation, those cells enter the irreversible process of endospore formation (Figure 2H) [31,34]. Interestingly, while high levels of Spo0A~P activate sporulation genes, they also curtail the expression of *sinI* by binding to multiple operators in the regulatory region of *sinI* (Figure 2I) [35]. Those operators imperfectly match the Spo0A~P consensus binding sequences and are only bound by Spo0A~P when its levels are high. This may not only explain the transition of matrix-producing into sporulating cells, but also why sporulating cells quickly exit matrix production and how these two cell types become mutually exclusive. Evidence from time-lapse fluorescent microscopy further supports this hypothesis [26].

In addition to Spo0A, other regulators such as DegU and the ComA-ComP two-component system are also shown to be important in activating matrix production and biofilm formation [38,39]. The complex regulatory network that controls biofilm formation has been reviewed extensively [22,40,41].

## 6. Control of Cell Differentiation by Bacterial Tyrosine Kinases

Differential gene regulation plays a key role in *B. subtilis* cell differentiation as we discussed above. However, cell differentiation can also be contributed to by post-translational regulations. One such regulation is mediated by bacterial tyrosine kinases (BY-kinase). In *B. subtilis*, there are two BY-kinases, PtkA and EpsB (also known as PtkB) [42]. Both pairs are involved in regulating cell differentiation [43,44,45,46]. The activities of PtkA and EpsB are regulated by their dedicated transmembrane modulators, TkmA and EpsA, respectively. Note that BY-kinases are present as single multidomain proteins in Gram-negative bacteria, whereas the kinase and the transmembrane modulator are two separate proteins in Gram-positive bacteria [42].

Published studies show that the EpsA-EpsB pair, whose genes are located in the *epsA-O* operon, is primarily involved in regulating post-translationally exopolysaccharide (EPS) biosynthesis. It is proposed that the membrane-anchored modulator EpsA is able to sense self-produced EPS as a signal probably through its extracellular domain [45]. Upon sensing the signal, EpsA interacts through its cytoplasmic c-terminus with the kinase EpsB and regulates autophosphorylation at its c-terminal tyrosine residue cluster of EpsB. EpsB phosphorylates its protein substrate, EpsE, a key glycosyltransferase involved in EPS biosynthesis, and thus regulates EpsE activities. Deletion of either *epsA* or *epsB* similarly resulted in a partial biofilm defect, suggesting that this BY-kinase pair has a regulatory function in biofilm formation [43]. Therefore, the secreted EPS and EpsA-EpsB-EpsE proteins constitute a regulatory feedback loop and a quorum-sensing like signaling pathway in regulating EPS biosynthesis and biofilm assembly [45].

Compared to the EpsA-EpsB, the function of TkmA-PtkA seems more promiscuous [47]. It has been demonstrated that PtkA phosphorylates and regulates a diverse set of substrate proteins involved in sugar synthesis, cell cycle control, DNA replication, fatty acid metabolism, and cell development [47,48,49,50]. TkmA and PtkA were also proposed to be involved in biofilm formation and sporulation [43,44,46]. Exactly how TkmA and PtkA contribute to biofilm formation is still unclear. In one published study, it was shown that although the *ptkA* gene deletion had an impact on the biofilm phenotype, mutagenesis of the tyrosine residues showed no impact on biofilm formation [44]. There are also studies suggesting that these two pairs of BY-kinases may crosstalk with each other, linking various environmental signals they sense and cell development and biofilm formation in *B.* subtilis [43,46]. In one study, it was shown that the modest biofilm defect caused by the *tkmA* gene deletion can be restored by complementation with a second copy of *epsA* expressed from the *tkmA* promoter, suggesting that the two kinase modulators may have an overlapping role in activating the EpsB kinase [46]. Overall, how the BY kinases regulate cell differentiation and development in *B. subtilis* deserves to be further explored in future studies.

## 7. Cell Differentiation Contributes to *B. subtilis* Environmental Prevalence

*B. subtilis* is widely present in the environment, and is one of the dominant species in the soil [51]. It is well known that the ability to produce stress-resistant dormant spores allows *B. subtilis* to survive better under harsh environmental conditions. Spore formation provides competitive advantages to *B. subtilis* over non-spore-forming soil bacteria. There are also other challenges that *B. subtilis* will face while living in the natural environment. Like many other soil-dwelling bacteria, dispersal is a major challenge for *B. subtilis* in order to prevail in the environment. Interestingly, certain differentiated cells in the biofilm may have found a way to collectively solve this problem by sticking out from the biofilm and/or the soil. In a mature *B. subtilis* biofilm, spores are found enriched at the tips of the so-called fruiting bodies. These are small visible aerial projections that rise from the surface of a biofilm (Figure 2D,E) [52]. Such a spatial distribution feature of spores is expected to greatly facilitate their dispersal in natural environments. One may postulate that in evolution, biofilm and endospore formation are interlinked. The biofilm serves as the structural framework to allow formation and proper spatial distribution of endospores; besides, it is a facilitator for spore dispersal when the biofilm ages or when environmental factors such as wind and rain trigger spore dispersal. It may be worth pointing out that aerial spore-containing structures evolved independently in a number of bacterial and eukaryotic species, through the process of convergent evolution, including *B. subtilis* [53].

Another major challenge for soil-dwelling microorganisms is migration in the soil. In the laboratory, *B. subtilis* is able to perform different types of motilities, including swimming, swarming, and twitching motilities [54,55,56,57]. In the natural environment, considering the semi-arid or arid nature of soil, one would expect a low possibility of constant swimming through liquid by bacteria. Swarming is defined as a population of motile cells migrating in synchrony on a semi-solid surface. However, the effectiveness of swarming could be limited since solid surfaces usually do not support robust swarming motility in *B. subtilis* [58]. So how does *B. subtilis* migrate in the soil and spread along, for example, the root surface? A recent study shows that *B. subtilis* is able to sense plant root-released sucrose and initiate a solid-surface motility through a novel mechanism [56]. Upon sensing sucrose, *B. subtilis* cells produce levan, a fructose-based polysaccharide. Levan then stimulates *B. subtilis* cell differentiation into hyperflagellated cells, along with strong production of a surface-wetting molecule, surfactin. This combination is necessary to trigger “hyper-swarming” in *B. subtilis* and allow the cells to migrate on solid surfaces. The differentiation into “hyper-swarmer” also significantly promotes competitive root colonization by *B. subtilis* in the soil. There are other examples of how cell differentiation contributes to *B. subtilis* as a dominant soil bacterial species. Thus, characterizing cell differentiation and its underlying mechanisms in *B. subtilis* will help us better understand how this bacterial species lives, functions, and prevails in the natural soil environments.

## 8. Conclusions

Biofilms are believed to be the predominant form of bacterial living in natural environments, and cell differentiation is a hallmark feature of biofilms. Biofilms thus serve as an excellent platform to investigate cell differentiation and generation of distinct cell types, and to understand its physiological and ecological importance in a more interactive context. In some cases, unique cell differentiation proceeds only during biofilm formation. Under these conditions, however, much still needs to be learned about cell differentiation in *B. subtilis*. Cell differentiation and biofilm formation are believed to a major contributor for the prevalence of *B. subtilis* and other related species in the natural environments. *B. subtilis* possesses various beneficial activities toward the environment and plant hosts. Since these beneficial activities largely rely on specialized or differentiated cell types of *B. subtilis*, it is thus important to better understand cell differentiation by this bacterial species in the natural environments.

## Data Availability

Data discussed in the review are all from and available through cited published literatures.
